# Supplementary material for: Beyond Patient Characteristics: A Narrative Review of Contextual Factors Influencing Involuntary Admissions in Mental Health Care
Source: Healthcare (Basel). 2023 Jul 9;11(14):1986. doi: 10.3390/healthcare11141986 (PMC10379438; doi:10.3390/healthcare11141986)
Supplement: Supplementary file 1 [file healthcare-11-01986-s001.zip › healthcare-2430202-supplementary.pdf]

Table S1: Overview of included articles

S/N	Title	Year of Publication	Country of study	Study Objective	Study design	Contextual factor	Main findings
1.	Mental health care in Athens: Are compulsory admissions in Greece a one-way road?[34]	2017	Greece	To estimate the frequency of compulsory admissions in a psychiatric hospital and to explore its underpinnings.	Cohort	Service-related	Patients who had been in touch with mental health services provided by the community before being admitted to a hospital were 6 times less likely to experience civil commitment compared to patients who had only interacted with mental health professionals during a previous hospital stay.
2	Involuntary Psychiatric Admissions and Development of Psychiatric Services as an Alternative to Full-Time Hospitalization in France [28]	2017	France	To assess whether the development of such alternatives in French psychiatric sectors was associated with a reduction in involuntary inpatient care, taking into account other factors that are potentially associated with involuntary admission.	Cross-sectional	Service-related	The level of development of alternatives to full-time hospitalization was significantly and negatively associated with involuntary admission. With every 10% rise in the level of advancement of alternatives, the chance of a patient being involuntarily admitted to full-time hospitalization decreased by 12%.
3	Involuntary Admission of Emergency Psychiatric Patients: Report from the Amsterdam Study of Acute Psychiatry [33]	2009	Netherlands	To determine which factors are associated with emergency compulsory admissions	Cohort	Service-related	Having over 14 outpatient interactions in the preceding year was linked to a decreased likelihood of being compulsorily admitted. Getting a referral from a general practitioner resulted in a lower risk of admission compared to referrals from the police or mental health services.
4	Poverty, poor services, and compulsory psychiatric admission in England [31]	2002	England	To examine the hypothesis that variation in rates of detention is related to social deprivation and also to the functioning of local mental health services.	Ecological	Service-related	A significant relationship between the rates of detention and social deprivation for both section types and for both genders, section type and service variables (delay in obtaining an acute bed, availability of visits after 10 p.m., and availability of visits to chronic patients more than twice weekly) were significant
5	Impact of social-psychiatric services and psychiatric clinics on involuntary admissions	2013	Germany	To identify factors influencing the voluntariness of admissions to psychiatric hospitals. Especially	Cross-sectional	Service-related	The quantity of supervised patients and home visits performed by social-psychiatric services indicated that in areas with a lower

	[35]			the impact of demographic factors of the region, characteristics of the psychiatric hospitals and characteristics of the psychosocial services was analyzed.			rate of involuntary admissions, a lesser number of clients required attention than in regions with a higher rate of involuntary admissions. The ratio of clients to staff in social-psychiatric services was approximately the same in regions with both low and high involuntary admission rates. However, regions with lower involuntary admission rates typically had a greater number of admissions from social-psychiatric services or general practitioners, on average.
6	The Nordic comparative study on sectorized psychiatry: rates of compulsory care and use of compulsory admissions during a 1-year follow-up [43]	1999	Multi-country	To perform a prospective investigation of organizational, clinical, and social predictors of being compulsorily admitted in seven Nordic catchment areas.	Cohort	Service-related	There was considerable disparity in the frequency of involuntary care among various services and Nordic countries. Analyzing three Swedish services that follow the same laws suggested that there may be a rural-urban trend, where the urban Stockholm service had higher rates of compulsory care. Furthermore, higher rates of compulsory care were connected with lower accessibility to psychiatric specialist services, as evidenced by the requirement for a formal referral process.
7	Involuntary admissions in accordance to the Mental Health Act (PsychKG) - what are the strongest predictors? [36]	2015	Germany	To compare voluntary and involuntary psychiatric admissions regarding personal, institutional, social-psychiatric and demographic characteristics.	Cohort	Service-related/Temporal	General practitioners were responsible for initiating the majority of voluntary admissions. Involuntary placement was frequently linked to certain admission characteristics, including admission during on-call hours (evenings and weekends) and emergency admission by the on-duty doctor. Clinics with a lower proportion of involuntary admissions reported being community-oriented, providing more crisis intervention resources such as home visits, close contacts, and low-threshold contact services. The sum of services provided was also associated with lower rates of

							involuntary admissions.
8	Does Assertive Community Treatment Reduce the Use of Compulsory Admissions?[29]	2017	Denmark	To test the hypothesis that Assertive Community Treatment (ACT) may have the quality to reduce the use of several type of coercion including compulsory admissions.	Cohort	Service-related	The ACT teams exhibited declining admission patterns, and there were noteworthy differences in trends for all admission types, including voluntary admission and admission based on danger criteria, compared to all other users of psychiatric services.
9	Trends in the use of the Italian Mental Health Act, 1979–1997[38]	2004	Italy	to establish whether the proportion of all psychiatric admissions that were compulsory changed significantly or remained stable over the 18 years after the new Mental Health Law was implemented in Italy	Cohort	Service-related/ Mental health legislation	Between 1979 and 1997, there was a significant reduction in the total number of psychiatric beds. During the same time period, the total number of compulsory admissions remained largely unchanged, but there was a decrease in the proportion of compulsory admissions made under the Act from 17.1% to 10.6%.
10	Differences in the use of involuntary admission across the Veneto Region: which role for individual and contextual variables?[37]	2016	Italy	To explore the relation between mental health services provision, characteristics of population, individual factors and involuntary admissions.	Cohort	Service-related	A higher prevalence rate of involuntary admission (IA) was predicted by higher ageing indices and rates of social workers. The contextual model explained more than 40% of the total variance.
11	Local psychiatric beds appear to decrease the use of involuntary admission: a case-registry study[42]	2014	Norway	To examine how a range of factors, including the organization of mental health services, affected rates of involuntary admission.	Cohort	Service-related	Patients from the deinstitutionalized system had a significantly greater risk of being involuntarily admitted. The deinstitutionalized system that did not have local beds available had the highest rate of involuntary admission.
12	Does mental health service integration affect compulsory admissions?[44]	2009	Netherlands	To evaluate the effect of varying level of service integration by naturalistic comparative analysis	Cohort	Service-related	Where mental healthcare was more integrated, services outcomes indicated improved results. Nonetheless, limited effects were observed, and it is possible that other factors beyond service integration may have played a more crucial role in preventing compulsory admissions.
13	Variation in requests to social services departments for assessment for compulsory	1993	England & Wales	Examines the reported frequency of requests for assessment for admission, and the variation in	Cross-sectional	Service-related	There was a correlation between requests for assessment and treatment order, as well as requests for emergency admission, and

	psychiatric admission [32]			the rate of requests according to the type of local authority and the extent of social deprivation.			greater social deprivation, but this relationship was only observed in London.
14	Association between provision of mental illness beds and rate of involuntary admissions in the NHS in England 1988-2008: ecological study [39]	2011	England	To examine the rise in the rate of involuntary admissions for mental illness in England that has occurred as community alternatives to hospital admission have been introduced	Ecological	Service-related	Over the same period, the rate of involuntary admissions per year in the NHS rose by over 60%, while the provision of mental illness beds dropped by more than 60%.
15	Variations in patterns of involuntary hospitalization and in legal frameworks: an international comparative study [13]	2019	Multi-country	To compare trends over time, and to explore whether variations in legislation, demographics, economics, and health-care provision might be associated with variations in involuntary hospitalization rates	Ecological	Service-related/Mental health legislation	There was no observed correlation between annual rates of involuntary hospitalization and any features of the legal framework. However, a larger number of beds was significantly associated with higher national rates of involuntary hospitalization.
16.	Involuntary admissions in Italy: the impact of Seasonality [53]	2016	Italy	To assess the prevalence of voluntary and involuntary admissions with particular reference to seasonality and clinical correlates in a sample of patients consecutively admitted to our psychiatric unit during a period of 24 months.	Cohort	Seasonal	The frequency of involuntary hospitalizations significantly increased during the seasonal changes, particularly in the spring/summer season with the highest peak in June. The seasonal changes have a significant impact on the development of psychiatric disorders, particularly in bipolar and related disorders. Therefore, seasonality may be an important factor influencing involuntary hospitalizations.
17.	The influence of sunlight exposure on hospitalization in emergency psychiatry [54]	2017	Italy	To evaluate the impact of photoperiod, and investigate the period of major/minor exposure to sunlight in a sample of patients consecutively admitted to our psychiatric unit during a period of 24 months,	Cohort	Seasonal	Patients admitted during the spring/summer seasons, which have a higher number of daylight hours, had a significantly higher occurrence of involuntary admissions compared to those admitted during other seasons.
18.	Clinical characteristics and outcomes associated with weekend admissions to psychiatric wards in Taiwan	2019	Taiwan	To investigate whether patients admitted to psychiatric wards in Taiwan on the weekend different and whether outcomes are	Cohort	Temporal	The proportion of compulsory admissions on the weekend was significantly higher than that on weekdays.

	[49]			different for patients admitted on the weekend?			
19	Clinical outcomes and mortality associated with weekend admission to psychiatric hospital [48]	2016	England	To investigate whether weekend admission to a psychiatric hospital is associated with worse clinical outcomes.	Cohort	Temporal	After adjusting for other demographic and clinical factors compulsory admission was less likely at the weekend than during the week.
20.	What is the role of meteorological variables on involuntary admission in psychiatric ward? An Italian cross-sectional study [55].	2020	Italy	To investigate the impact of meteorological variables and other indexes in a large sample of hospitalized patients, focusing on subjects who were involuntarily admitted	Cohort	Seasonal	The study found that there were significantly higher maximum and medium temperatures, as well as humidex, during involuntary admissions compared to voluntary admissions. Logistic regression analysis showed that maximum temperature and humidex remained significantly associated with involuntary admission.
21.	Relationship Between Time of Admission, Help-Seeking Behavior, and Psychiatric Outcomes: “From Dusk Till Dawn” [46]	2022	Switzerland	To investigate the relationship between time of admission, patients' demographic and clinical profile, and treatment outcomes.	Cohort	Temporal	There were fewer compulsory admissions during office hours and daytime. The number of compulsory admissions increased gradually from dusk until dawn and reached its peak around 5-6 a.m. The increase in compulsory admissions was more noticeable on weekends.
22.	Compulsory Admission to Psychiatric Wards-Who Is Admitted, and Who Appeals Against Admission? [47]	2019	Switzerland	to examine if specific sociodemographic and clinical characteristics are associated with involuntary admission and with an appeal against the compulsory admission order.	Cohort	Temporal	Patients who were admitted involuntarily were more likely to be admitted during nighttime or on weekends, as compared to patients who were admitted on a voluntary legal status.
23.	Predictors of involuntary hospitalizations to acute psychiatry [45]	2013	Norway	To compare involuntary referred with voluntary referred patients	Cross-sectional	Temporal	Patients who were admitted involuntarily showed a statistically significant higher likelihood of being admitted during the evening and night shifts.
24.	Involuntary Psychiatric Examinations for Danger to Others in Florida After the Attacks of September 11, 2001 [71]	2005	United States	To test the general hypothesis that the threshold for what constitutes sufficient danger to warrant involuntary treatment declines in a community after widely reported or witnessed violent behavior	Cross-sectional	Impactful event	In the aftermath of September 11, 2001, law enforcement officers in Florida brought in a greater number of individuals, both men and women, for psychiatric evaluations in order to prevent potential harm to others. This was a departure from the usual historical patterns

				using interrupted time series design			and the number of people referred to mental health professionals.
25	Rates of unemployment and incidence of police-initiated examinations for involuntary hospitalization in Florida [60]	2006	United States	To test the hypothesis that contraction of regional economies affects the incidence of involuntary admissions to psychiatric emergency services by reducing community tolerance for persons perceived as threatening to others	Cross-sectional	Impactful event	In periods of unemployment, law enforcement officers referred approximately 309 more men for neglect compared to historical presentation rates and the number of individuals referred by mental health professionals and police.
26	Medicaid Cutbacks and State Psychiatric Hospitalization of Patients With Schizophrenia [61]	2011	United States	to examine state psychiatric hospital utilization among persons with schizophrenia who maintained or lost Medicaid coverage.	Cohort	Impactful event	Analyses controlling for age, gender, race-ethnicity, Medicaid eligibility, and Medicare coverage showed that persons who maintained Medicaid coverage had little change in state psychiatric hospitalization (Involuntary admissions), whereas utilization increased markedly over time for those who lost Medicaid coverage. There were minimal differences in the use of general hospital psychiatric units. It was generally observed that loss of Medicaid coverage occurred before the increase in hospitalizations.
27	Association of Cost Sharing With Mental Health Care Use, Involuntary Commitment, and Acute Care [63]	2017	Netherlands	To examine the association of higher patient cost sharing with mental health care use and downstream effects, such as involuntary commitment and acute mental health care use.	Cohort	Impactful event	An increase in out-of-pocket expenses was linked to decreased utilization of mental health care, leading to cost savings overall, but also resulting in increased expensive use of involuntary commitment and acute care, especially among those with psychotic disorder or bipolar disorder.
28	Patterns of adult and youth inpatient admissions before and after the COVID-19 pandemic in a psychiatric ward: an observational study [64]	2022	Portugal	To understand whether COVID affected the main characteristics of psychiatric hospitalizations, measured by admissions in a Portuguese acute psychiatric ward.	Pre-post	Impactful event	Although there was a decrease in the overall number of patients admitted, severe conditions such as compulsory admissions and diagnosis of schizophrenia did not show a similar reduction.
29	Psychiatric hospitalization rates in Italy before and during	2020	Italy	To assess admission rates to seven General Hospital Psychiatric	Pre-Post	Impactful event	Between February 21 and March 31, 2020, which marked the beginning of the COVID-

	COVID-19: did they change? An analysis of register data [67]			Wards (GHPWs) located in the Lombardy Region in the 40 days after the start of Coronavirus disease 2019 (COVID-19) epidemic, compared to similar periods of 2020 and 2019.			19 epidemic in Italy, there was a significant decline in psychiatric admission rates specifically for voluntary admissions, compared to a similar 40-day period prior to February 21, as well as two 40-day periods in 2019. However, there was no noticeable reduction in admission rates for involuntary admissions during the same time period.
30	Impact of the COVID-19 Pandemic on Psychiatric Admissions to a Large Swiss Emergency Department: An Observational Study [65]	2021	Switzerland	To investigate differences in admissions at a Swiss psychiatric ED from 1 April to 15 May during a “pandemic-free” period in 2016 and a “during-pandemic” period in 2020.	Pre-Post	Impactful event	During the COVID-19 pandemic, patients who were admitted to the psychiatric emergency department were significantly more likely to be hospitalized involuntarily after their psychiatric consultation compared to a period without a pandemic.
31	The effect of COVID-19 lockdown on psychiatric admissions: role of gender [66]	2021	United Kingdom	To examine whether the first UK lockdown changed the demographics of patients admitted to psychiatric hospitals (to include gender, legality, route of admission and diagnoses), independent of seasonal variation	Pre-Post	Impactful event	Although there was a decrease in the overall number of admissions, there was a consistent and statistically significant rise in the percentage of admissions that resulted from formal sectioning throughout all six months of 2020. This trend was observed in both male and female patients.
32	Reasons behind the rising rate of involuntary admissions under the Mental Health Act (1983): Service use and cost impact. [40]	2020	United Kingdom	To explore potential impactful events that may have contributed to unexpected changes in rates of involuntary psychiatric hospitalization	Cohort	Impactful event/Service-related/Mental health Legislation	Admission rates have steadily increased year after year since the conclusion of the National Service Framework for Mental Health and the start of austerity measures in 2010/11.
33	Why did China’s mental health law have a limited effect on decreasing rates of involuntary hospitalization? [74]	2022	China	To examine the rates and correlates of IH in a large psychiatric hospital in Guangzhou from 2014 to 2017 after the implementation of MHL and a structured assessment of the need for IH.	Cohort	Mental health legislation	Following the implementation of a structured assessment of need for involuntary hospitalization, there was a noticeable but minor rise in rates of inpatient hospitalization in China. The legislation appeared to have been unsuccessful in reducing inpatient hospitalization due to a shortage of available hospital beds and the impact of cultural stigma.
34	Changing patterns in emergency involuntary	2008	Netherlands	To investigate changes in demographic or diagnostic	Cohort	Mental health legislation	After adjusting for changes in age and gender distribution, there was a rise in the

	admissions in the Netherlands in the period 2000–2004 [75]			characteristics of committed patients in the Netherlands and whether there were changes in dangerousness criteria related to danger to the public, used to justify commitment.			number of commitments between 2000 and 2004. The most significant increase was observed in the 'arousing aggression' criterion for dangerousness.
35	Epidemiology of involuntary placement of mentally ill people across the European Union ill people across the European Union [76]	2004	Multi-country	To describe and compare the legal frameworks and routine procedures of compulsory admission and involuntary treatment of all European Union Member States in a systematic and standardized manner	Cohort	Mental health legislation	Member States that required the presence of a legal representative had significantly lower rates of compulsory admission and showed a tendency towards lower rates of compulsory admission overall. This was the only procedural feature among the assessed legal characteristics that effectively differentiated between clusters of Member States based on their legal regulations for involuntary placements of mentally ill individuals.
36	Clinical and socio-demographic characteristics associated with involuntary admissions in Switzerland between 2008 and 2016: An observational cohort study before and after implementation of the new legislation [73]	2019	Switzerland	To describe characteristics of a cohort of involuntarily compared to voluntarily admitted patients regarding clinical and socio-demographic characteristics before and after implementation of the new legislation	Cohort	Mental health legislation	The implementation of the new legislation in January 2013 was significantly associated with a reduced risk of Involuntary Admission.
37	North Carolina Resident Psychiatrists Knowledge of the Commitment Statutes: Do They Stray from the Legal Standard in the Hypothetical Application of Involuntary Commitment Criteria? [77]	2010	United States	To examine North Carolina (NC) resident psychiatrist's knowledge of commitment statutes and their willingness to involuntarily admit hypothetical patients who do not meet statutory criteria	Cross-sectional	Staff factors	North Carolina (NC) resident psychiatrists' decision to involuntary admit patients was not affected by their understanding of commitment statutes. However, the decision was influenced by the need for transportation, particularly in cases of substance abuse patients. Psychiatrists who deemed secure transportation to the hospital necessary were more likely to commit the patient.
38	Psychiatrists' attitudes toward involuntary hospitalization	2004	United States	To examine whether psychiatrists' attributions of responsibility for	Cross-sectional	Staff factors	The study did not find any significant correlation between attributions of

	[78]			mental illnesses affect their decisions about involuntary hospitalization			responsibility and the decision to commit individuals with mental illness. Psychiatrists surveyed showed reluctance to involuntarily hospitalize patients with mental illness in the absence of immediate harm.
39	US psychiatrists' beliefs and wants about involuntary civil commitment grounds [86]	2006	United States	To discover some of the apparent sources of psychiatric norms about commitment grounds.	Cross-sectional	Staff factors	The study found that the respondents' level of support for different commitment grounds was strongly linked to their understanding of the laws in their state. This suggests that the respondents had internalized the norms and preferences of their state's commitment grounds.
40	Risk assessment and its influencing factors of involuntary admission in patients with mental disorders in Shanghai, China [84]	2021	China	To explore the application of risk criterion in involuntary admission cases; especially risk assessed by psychiatrists at admission and its influencing factors.	Cross-sectional	Staff factors	About half of patients were involuntarily admitted due to behaviors that posed a risk to themselves or others. However, nearly 10% of patients were involuntarily admitted despite no documentation of risk assessment checklists being completed.
41	Psychiatrists' opinions about involuntary civil commitment: Results of a national survey [87]	2007	United States	To update knowledge about the opinions of psychiatrists through a survey based on a national random sample of members of the American Psychiatric Association	Cross-sectional	Staff factors	The survey respondents provided responses that were frequently inaccurate when it came to their knowledge of the grounds for commitment in their states, as well as their understanding of whether involuntary outpatient commitment was allowed in their respective states.
42	To commit or not to commit: the psychiatry resident as a variable in involuntary commitment decisions [81]	2006	United States	To study whether psychiatry residents’ personal variables (such as age, gender, level of training, previous experience with patient suicide, or lawsuits) and their temperamental predispositions have an impact on their decisions to seek involuntary commitment.	Cross-sectional	Staff factors	The findings indicated a notable correlation between the inclination of psychiatrists to take risks and their level of education, and their choices to refrain from pursuing involuntary hospitalization for the patients in the five simulated clinical scenarios.
43	Impact of Psychiatrists' Qualifications on the Rate of Compulsory Admission [83]	2013	Switzerland	To assess the impact of this *change of procedure* on the proportion compulsory admissions<br><br>*Since October 2006, only	Pre-post	Staff factors	From October 2006 onwards, patients who were hospitalized were less likely to be admitted involuntarily compared to the period before October 2006.

				certified psychiatrists are authorized to require a compulsory admission to the facility, while before all physicians were, including residents			
44	Involuntary admission for psychiatric treatment: Compliance with the law and legal considerations in referring physicians with different professional backgrounds [82]	2019	Switzerland	To examine whether legal considerations differed according to the physicians` professional backgrounds and attitudes towards coercion in general.	Cross-sectional	Staff factors	Around a third of the participants reported that they were unable to meet certain legal requirements aimed at promoting patient autonomy during involuntary admission, citing the patients' clinical condition as the most common reason for omitting procedures such as hearing the patients' views, providing information about next steps, and the right to appeal. Psychiatrists demonstrated greater familiarity with the legal basis for involuntary admission compared to other physicians, and the physicians more frequently viewed coercion as a form of treatment.
45	Involuntary psychiatric admission: Characteristics of the referring doctors and the doctors' experiences of being pressured [85]	2015	Norway	To examine characteristics (gender, work place, work experience, prior knowledge of referred patient) of Genera Practitioners (GPs) that admitted patients involuntarily to a psychiatric hospital, and to examine how the GPs assessed this process	Cross-sectional	Staff factors	Almost half of the respondents stated that they had felt pressured/advised to refer the patient involuntarily to hospital.<br>The pressure/advice to refer/refer involuntarily came from — in order of importance — the health service, the patient’s family and the police
46	Clinicians’ decision making about involuntary commitment [41]	1998	United States	To examine Clinicians' decision making about involuntary commitment, with a focus on the effects of patient and clinician characteristics and bed availability on decisions to detain patients, the first step in involuntary commitment.	Cross-sectional	Staff factors	Several factors were discovered to impact the decision to involuntarily commit patients, such as the clinician's inclination to detain patients and the accessibility of beds for detention.
47	Does physician compensation for declaration of involuntary	2020	Canada	To examine whether in a government-funded health care	Cross-sectional	Staff factors	The probability of being involuntarily admitted rose when there was a 10%

	status increase the likelihood of involuntary admission? A population-level cross-sectional linked administrative database study[88]			system, physician payments for filling forms related to an involuntary psychiatric hospitalization were associated with the likelihood of an involuntary admission.			increase in the absolute percentage of financially reimbursed forms at the Emergency Department.
48	Potentially Dangerous Behavior in the Mentally Ill: Attitudes of Journalists and Medical Students Toward Compulsory Admission [90]	2002	Austria	To investigate what medical students and journalists consider legitimate conditions for compulsory admission	Cross-sectional	Public attitude & stigma	The most commonly cited reasons for compulsory admission among both groups were instances of violence towards others. Approximately one-third of the participants in each group also believed that ongoing neglect warranted involuntary commitment. Students were more likely than journalists to endorse civil commitment in cases involving suicide attempts and violent behavior. Medical students who had personal experience with individuals with mental illness were significantly more likely to advocate for coercive measures in cases of suicide attempts.
49	Public attitude to compulsory admission of mentally ill people [94]	2002	Switzerland	Assessing the public attitude to the compulsory admission of the mentally ill in Switzerland, and analyzing the influence of demographic, psychological and sociological factors	Cross-sectional	Public attitude & stigma	The majority of the respondents were in favor of compulsory admission for those with mental illness. However, factors such as level of education, negative stereotypes, and living in French-speaking regions of Switzerland were found to be associated with a higher likelihood of supporting mandatory admission.
50.	Public attitude towards restrictions on persons with mental illness in greater Hanoi area, Vietnam [93]	2018	Vietnam	To explore whether socio-demographic factors affect public attitudes toward restrictions on mentally ill people in Hanoi, Vietnam.	Cross-sectional	Public attitude & stigma	The majority of participants in the study supported two restrictions for individuals with mental illness: mandatory hospitalization under specific circumstances and revocation of their right to vote in parliamentary elections. Men were found to be less likely than Vietnamese women to approve of mandatory hospitalization. Most participants agreed on three conditions that warrant involuntary admission: suicidal

							behavior, violent behavior towards others, and self-neglect and isolation due to persecutory delusions.
51.	French lay people's views regarding the acceptability of involuntary hospitalization of patients suffering from psychiatric illness [92]	2012	France	To understand how lay people and health professionals in France judge the acceptability of hospitalizing a psychiatric patient against his will	Cross-sectional	Public attitude & stigma	Most of the participants in the study believed that involuntary hospitalization is appropriate in specific situations, particularly when the patient poses a danger to others, in accordance with French regulations.
52.	Public attitudes towards involuntary admission and treatment by mental health services in Norway [91]	2017	Norway	To examine the attitudes in a representative sample of Norway's population towards the use of involuntary admission and treatment, and under which circumstances does the general public consider compulsory treatment to be justified in the Norwegian mental health care services.	Cross-sectional	Public attitude & stigma	The majority of Norwegian adults endorse the current laws and practices related to involuntary admission and compulsory treatment in mental health services. The most significant support for involuntary treatment was seen in cases where the patient was unable to take care of themselves, such as those with depression and suicidal thoughts. People with lower levels of education were more likely to have a favorable view of involuntary admission and treatment.
53.	Attitudes of the German public to restrictions on persons with mental illness in 1993 and 2011 [96]	2014	Germany	To explore to what extent these achievements are reflected in changes of public attitudes towards restrictions on mentally ill people.	Cross-sectional	Public attitude & stigma	There are two contrasting trends in public opinions regarding limitations on individuals with mental illness. On one hand, there is a general shift towards more liberal views on patients' rights. However, there is also an inclination towards restricting the freedom of patients in cases of nonconformist behavior.
54.	Approval of Coercion in Psychiatry in Public Perception and the Role of Stigmatization [95]	2021	Switzerland	To examine the approval for coercive measures in psychiatry by the general public, and to explore its relation with person- and situation-specific factors as well as with stigmatization.	Cross-sectional	Public attitude & stigma	The acceptance of coercion in psychiatric treatment was found to be positively correlated with perceived dangerousness and perceived effectiveness of the treatment. Familiarity with psychiatric treatment, particularly having a friend who has undergone treatment, was associated with a decreased acceptance of coercion. The gender of the hypothetical patient, the

gender of the participant, and the desire for social distance were not significant factors in the general acceptance of coercion.
